# MiR-21 Enhances Melanoma Invasiveness via Inhibition of Tissue Inhibitor of Metalloproteinases 3 Expression: In Vivo Effects of MiR-21 Inhibitor

**DOI:** 10.1371/journal.pone.0115919

**Published:** 2015-01-14

**Authors:** Sara E. Martin del Campo, Nicholas Latchana, Kala M. Levine, Valerie P. Grignol, Ene T. Fairchild, Alena Cristina Jaime-Ramirez, Thao-Vi Dao, Volodymyr I. Karpa, Mary Carson, Akaansha Ganju, Anthony N. Chan, William E. Carson III

**Affiliations:** 1 Department of Surgery, The Ohio State University, Columbus, Ohio, United States of America; 2 Comprehensive Cancer Center, The Ohio State University, Columbus, Ohio, United States of America; 3 Department of General Pediatrics, Nationwide Children’s Hospital, Columbus, Ohio, United States of America; 4 Department of Neurological Surgery, The Ohio State University, Arthur Giangiacomo James Cancer Hospital and Richard Jack Solove Research Institute, Columbus Ohio, United States of America; 5 School of Medicine, Wright State University, Dayton, Ohio, United States of America; 6 Department of Molecular Virology, Immunology and Medical Genetics, The Ohio State University, Arthur Giangiacomo James Cancer Hospital and Richard Jack Solove Research Institute, Columbus Ohio, United States of America; 7 School of Medicine, Northeast Ohio Medical University, Rootstown, Ohio, United States of America; University of Illinois at Chicago, UNITED STATES

## Abstract

Metastatic melanoma is the most aggressive form of this cancer. It is important to understand factors that increase or decrease metastatic activity in order to more effectively research and implement treatments for melanoma. Increased cell invasion through the extracellular matrix is required for metastasis and is enhanced by matrix metalloproteinases (MMPs). Tissue inhibitor of metalloproteinases 3 (TIMP3) inhibits MMP activity. It was previously shown by our group that miR-21, a potential regulator of TIMP3, is over-expressed in cutaneous melanoma. It was therefore hypothesized that increased levels of miR-21 expression would lead to decreased expression of TIMP3 and thereby enhance the invasiveness of melanoma cells. miR-21 over-expression in the melanoma cell lines WM1552c, WM793b, A375 and MEL 39 was accomplished via transfection with pre-miR-21. Immunoblot analysis of miR-21-overexpressing cell lines revealed reduced expression of TIMP3 as compared to controls. This in turn led to a significant increase in the invasiveness of the radial growth phase cell line WM1552c and the vertical growth phase cell line WM793b (p < 0.05), but not in the metastatic cell lines A375 or MEL 39. The proliferation and migration of miR-21 over-expressing cell lines was not affected. Reduced expression of TIMP3 was achieved by siRNA knockdown and significantly enhanced invasion of melanoma cell lines, mimicking the effects of miR-21 over-expression. Treatment of tumor cells with a linked nucleic acid antagomir to miR-21 inhibited tumor growth and increased tumor expression of TIMP3 in vivo in 01B74 Athymic NCr-nu/nu mice. Intra-tumoral injections of anti-miR-21 produced similar effects. This data shows that increased expression of miR-21 enhanced the invasive potential of melanoma cell lines through TIMP3 inhibition. Therefore, inhibition of miR-21 in melanoma may reduce melanoma invasiveness.

## Introduction

The incidence of melanoma is increasing faster than any other cancer in the United States. In 2013, it is estimated that 76,690 new cases of melanoma will be diagnosed and that there will be 9,480 deaths due to melanoma [1]. Surgery can be curative for early stage lesions with 5-year survival rates of 92–99% for Stage 1A/B melanoma [2]. However, when metastatic disease is present, 5-year survival rates can be as low as 10%. Cytotoxic chemotherapy for metastatic melanoma exhibits modest response rates of less than 20%, and while targeted therapies show promise, toxicities and the development of resistance are problematic [3]. Understanding the mechanisms of invasion and metastasis of this disease is critical to identifying new therapeutic targets.

In order for metastasis to occur, changes in cytoskeletal organization and altered contacts with the extracellular matrix (ECM) are necessary to increase cancer cell motility [4]. Degradation of ECM by matrix metalloproteinases (MMPs) is involved in promoting tumor growth, invasion and angiogenesis [5], and MMPs have been found to be upregulated in melanoma [6]. Tissue inhibitor of metalloproteinases-3 (TIMP3) is a member of the protein family that binds metalloproteinases and other proteolytic enzymes to reduce their activity [7]. High expression levels of TIMP3 have been associated with decreases in invasion due to decreased extracellular matrix degradation, decreased angiogenesis due to the prevention of VEGF binding to VEGFR2, and increased apoptosis [8]. Conversely, decreased TIMP3 expression has been observed in a variety of malignancies and has been correlated with aggressiveness in cancers arising in the thyroid, breast, prostate and lung, which supports a role for TIMP3 as a tumor suppressor via its ability to inhibit MMPs [9–13]. We propose that reversing this loss of TIMP3 expression would lead to a less invasive phenotype.

MicroRNAs (miRs) are a class of small, non-coding RNAs that negatively regulate protein translation by binding to the mRNA three prime untranslated region (3’UTR), which results in mRNA degradation, or repression of translation [14, 15]. Studies have shown miRs to be differentially expressed in solid and hematologic malignancies, including melanoma. miRs affect multiple tumorigenic processes including angiogenesis, cell cycle control, cellular adhesion, and apoptosis [16, 17]. Our group previously identified miR-21 as being over-expressed in primary cutaneous melanomas as compared to benign nevi, suggesting that miR-21 may play a role in melanoma pathogenesis [18]. TIMP3 has been identified as a putative target and has been shown to be decreased in response to miR-21 over-expression in cholangiocarcinoma and glioma [19, 20]. Co-transfection of an anti-miR-21 oligonucleotide, a renilla luciferase vector, and a pGL3-TIMP3 vector led to an increase in luciferase activity in breast cancer cell lines, indicating direct interaction of miR-21 on TIMP3 expression at the translational level [21]. However, the specific functions of this miR in melanoma have yet to be elucidated.

In this report, the effect of increased miR-21 expression on melanoma cell line behavior was evaluated. Cell lines derived from different stages of melanoma development exhibited increased invasion and decreased TIMP3 protein expression when miR-21 was over-expressed. Decreased TIMP3 expression recapitulated this increase in melanoma cell line invasion. Finally, murine models revealed that a miR-21 antagonist could inhibit melanoma tumor growth.

## Materials and Methods

### Ethics Statement

This study was performed in strict accordance with the recommendations in the Guide for the Care and Use of Laboratory Animals of the National Institutes of Health. The protocol was approved by the Ohio State University’s Institutional Animal Care and Use Committee (IACUC) [Protocol#2009A0179]. All surgery was performed under isoflurane anesthesia, and every effort was made to minimize suffering.

### Cell Lines

The human radial growth melanoma cell line WM1552c and the human vertical growth melanoma cell line WM793b were provided by Dr. M. Herlyn (Wistar Institute, Philadelphia, PA) and cultured as previously described [22]. The human metastatic melanoma cell line MEL 39 was a gift from Soldano Ferrone (Harvard Medical School, Boston, MA) and cultured as previously described [23]. The A375 human metastatic melanoma cell line was obtained from American Type Cell Culture Collection (ATCC, Manassas, VA).

### Oligonucleotide Transfections

Cells were plated on 60 mm^2^ plates at a density of 1 × 10^6^ cells per plate. When the cells were 70–80% confluent, they were transfected with pre-miR-21 or control pre-miR (Ambion, Austin, TX) using *Trans*IT TKO transfection reagent (Mirus Bio, Madison, WI). Twenty-four hours post-transfection, cells were collected for use in all assays. Tissue inhibitor of metalloproteinase 3 (TIMP3)-specific small-interfering RNA (siRNA) and negative control constructs were purchased from Santa Cruz Biotechnology, Inc (Santa Cruz, CA). The sequences for each oligo are as follows:

Ambion Control miR: AGUACUGCUUACGAUACGGTT
Ambion Pre-miR miRNA Precursor: hsa-miR-21–5p: (UAGCUUAUCAGACUGA UGUUGA)
Ambion Anti-miR miRNA Inhibitor: hsa-miR-21–5p (UAGCUUAUCAGACUGAU GUUGA)
Santa Cruz Control siRNA (UUCUCCGAACGUGUCACGU)
Santa Cruz TIMP3 siRNA (h2) sc-44331 consists of a pool of three siRNAs with sequences:
  - GGUAUCACCUGGGUUGUAAtt
  - GAACCUGUAUUCCUCUUCUtt
  - GAGAGUAGGUGAUAAUGUAtt

### Transfection Efficiency

Transfection efficiency was assessed using a FAM labeled miR construct (Ambion, Austin, TX) with the same transfection method above. Following harvest, 5×10^4^ cells were plated on cover slips overnight before staining with DAPI. Confocal images were captured using an Olympus FV1000-Spectral microscope equipped with a PLANFLN 40x oil-immersion objective lens (N.A. 1.3) (S1 Fig.). All imaging for each fluorescence signal was performed under identical detector settings.

### RNA extraction and Real-Time PCR

Total RNA from cells was isolated using TRIzol reagent (Invitrogen) as per the manufacturer’s recommendations for both mRNA and miR analyses. For analysis of miR expression, Fast Real-Time PCR analyses were carried out using TaqMan miR assays (Applied Biosystems) according to the manufacturer’s protocol. Relative expression was normalized to RNU6B, a small ubiquitous RNA. Relative levels of TIMP3 mRNA were examined using Fast Real-Time PCR and normalized to levels of Beta Actin. All reagents, primers and probes were obtained from Applied Biosystems (Foster City, CA). Gene and miR expression levels were quantified using the ABI Prism 7900HT Sequence Detection System (Applied Biosystems). Comparative Real-Time PCR, using the Ct method, was performed in triplicate, including no-template controls [24]. Expression was calculated as fold change (2^-ΔΔCt^) compared with the control pre-miR-transfected cells or siRNA negative control‑transfected cells.

### Proliferation Assay

Cell proliferation was measured as absorbance at 570 nm using the 3-(4,5-Dimethylthiazol-2-yl)-2,5-Diphenyltetrazolium Bromide (MTT) Cell Proliferation Assay kit according to the manufacturer’s instructions (ATCC, Manassas, VA). All assays were performed in triplicate.

### Migration Assays

Cells were plated on 60 mm^2^ plates at a density of 1 × 10^6^ cells per well. When the cells were 70–80% confluent, they were transfected with pre-miR-21 or control miR-21. Twenty-four hours post-transfection, cells were collected and counted. Radius 24-well Cell Migration Assays (Catalog # CBA-125-ECM, Cell Biolabs, Inc., San Diego, CA) were used, following the manufacturer’s instructions. Each well contains a circular 680 μm-diameter gel spot to which cells do not attach. Each plate has a row of 6 wells coated in Collagen I, a row coated in Fibronectin, a row coated in Laminin I, and an uncoated row. A375 cells were seeded at a density of 0.5 × 10^6^ cells per milliliter (mL), and WM1552c, WM793b, and MEL 39 cells were seeded at a density of 0.75 × 10^6^ cells per mL. The plated cells were incubated at 37°C for 16 hours to 80% confluence. The cells were treated with 10 µg/mL Mitomycin C (Sigma Aldrich, St. Louis, MO) for 3 hours to inhibit cell proliferation [25]. The Radius gel spot was removed according to the manufacturer’s protocol and media was changed to 10% serum. Wounds were monitored, and digital images of gap closure were taken using an Olympus IX50 inverted microscope at 10X magnification (1280 × 1024 pixels) (Olympus Imaging America, Inc., Center Valley, PA). Areas of gap closure were measured using ImageJ processing software (National Institutes of Health, Bethesda, MD). Data is shown as percentage of the original wound area.

### Invasion Assays

Matrigel invasion assays were conducted according to manufacturer’s instructions (BD Biosciences, San Jose, CA). Briefly, 5 × 10^4^ transfected cells in media containing 2% FBS were plated in duplicate on Transwell filters coated with or without Matrigel. The lower compartments of the invasion chambers contained media with 10% FBS as chemoattractant. After an 18-hour incubation at 37°C, cells remaining on the upper surface of the filter were removed, and the cells that migrated through the filter were fixed, stained, and counterstained with Dip Quick Stain Kit (Jorgensen Laboratories, Inc., Loveland, CO). Photographs were taken of five 20X fields for each filter on an EVOS XL digital inverted microscope and cell numbers were enumerated from the images (2048 × 1536 pixels) (Advanced Microscopy Group, Bothell, WA). Data is expressed as the percent invasion through the Matrigel matrix by calculating the ratio of the mean number of cells that invaded through the Matrigel matrix to the mean number of cells that migrated through the control insert.

### Immunoblot Analysis

Twenty-four hours post-transfection, cells were collected and lysed by standard procedure in radioimmunoprecipitation assay buffer (Sigma Aldrich, St. Louis, MO) containing protease inhibitor and phosphatase inhibitor cocktails (Thermo Fisher Scientific, Inc., Waltham, MA). Proteins were separated by SDS-PAGE, transferred to nitrocellulose or polyvinylidene difluoride filters and probed with antibodies specific for TIMP3, programmed cell death protein 4 (PDCD4), tropomyosin-1 (TM1), (Santa Cruz Biotechnology, Santa Cruz, CA), phosphatase and tensin homolog (PTEN) (Cell Signaling Technology, Danvers, MA), or β-actin (Sigma-Aldrich, St. Louis, MO). Following incubation with the appropriate horseradish peroxidase-conjugated secondary antibodies, immune complexes were detected using the Pierce ECL Western Blotting Substrate (Thermo Fisher Scientific, Inc., Waltham, MA). β-actin was used to confirm equal loading.

### Flow Cytometry

TIMP3 protein expression was analyzed in Mel39 cells following overnight transfection with a pre-miR 21 or a control miR (25 uM) oligonucleotide prior to harvest. Cells were then incubated with PE-anti-TIMP3 or isotype control Ab, washed and fixed in 1% formalin. A total of 10,000 cells were analyzed on a LSRII flow cytometer.

### *In vivo* Studies

LNA oligonucleotides against miR-21 and a negative control oligonucleotide were obtained from Exiqon, Inc. (Woburn, MA). For the experiments of the *in vitro* transfected A375 cells, knockdown oligonucleotides were transfected using *Trans*IT TKO transfection reagent (Mirus Bio, Madison, WI) into A375 cells at a final concentration of 50 nM each. After 24 hours from transfection, cells were collected, and miR-21 expression was analyzed by Real-Time PCR to verify effective miR knockdown. At the same time point, 2 × 10^6^ cells were resuspended in PBS and subcutaneously injected into the right flank of 6 week old female 01B74 Athymic NCr-nu/nu mice (n = 4 for the control LNA group and n = 6 for the anti-miR-21 LNA group) (Frederick National Library—NCI, Frederick, MD). Tumor growth was monitored by caliper measurement three times a week for 3 weeks.

For the *in vivo* antagomir treatments, 6 week old female 01B74 Athymic NCr-nu/nu mice (Frederick National Library—NCI, Frederick, MD) were subcutaneously injected in the right flank with 2 × 10^6^ cells A375 cells. After the tumors had reached an average volume of 100 mm^3^, the tumors were directly injected with 50 μL of PBS alone, or 50 μL of PBS and diluted *Trans*IT TKO transfection reagent containing 500 nM control LNA or 500 nM anti-miR-21 LNA (n = 5 per group). Tumors were treated intra-tumorally at days 0, 4, 7, and 11, for a total of 4 injections per tumor. Tumor growth was monitored by caliper measurement three times a week for 4 weeks.

Tumor volume was calculated as follows: V = L × l^2^ × 0.5, where L and l represent the larger and the smaller tumor diameter, respectively. At the end of each study, animals were sacrificed, and tumors were collected with a portion fixed in formalin for immunohistochemistry. Animals were housed by the University Laboratory Animal Resources according to institutional guidelines, and all experiments were approved by the Institutional Animal Care and Use Committee (The Ohio State University, Columbus, OH).

### Immunohistochemistry

The tumors were excised, fixed in a 10% formaldehyde solution, embedded in paraffin, and cut into slices for staining. A set of slides was stained with haematoxylin eosin for the morphological study and for the count of mitosis. To evaluate the mitotic index, three fields at 400X magnification were randomly selected, and the number of mitoses was counted. Another set of slides was stained with the goat IgG polyclonal antibody to TIMP3 (Santa Cruz Biotechnology, Santa Cruz, CA) or with normal goat IgG as a control (Sigma-Aldrich, St. Louis, MO). Brown, granular intracellular staining was considered to be positive for TIMP3. Immunostaining was scored on tumor tissue sections from each mouse by an independent pathologist who was blinded to group identity.

### Statistical Analysis

Statistical significance of differences between groups was analyzed by unpaired Student’s *t* test, and p ≤ 0.05 was considered to be statistically significant.

## Results

### Increased miR-21 activity does not affect melanoma proliferation

**Figure 1:**
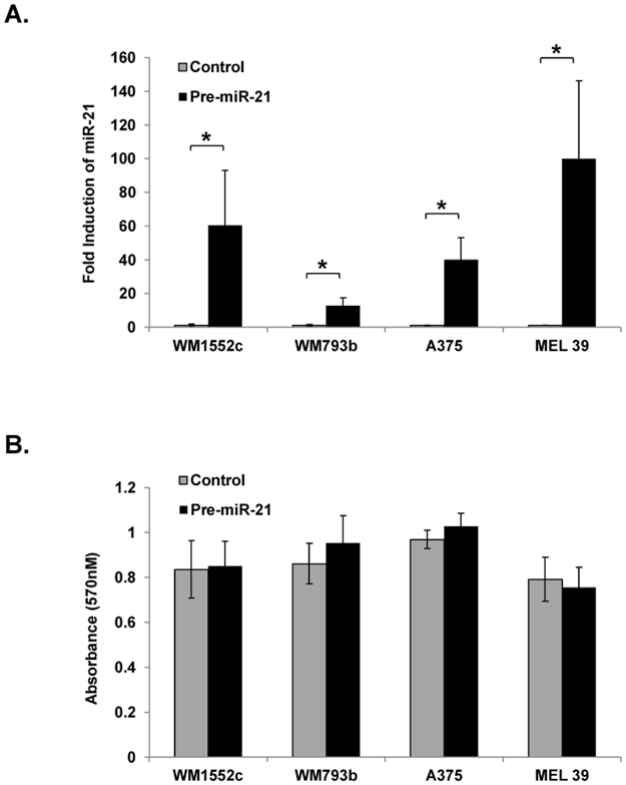
Mature miR-21 is overexpressed in cell lines transfected with pre-miR-21. Real-Time PCR was used to determine the expression of mature miR-21 in four human melanoma cell lines 24 hours post-transfection with control pre-miR or pre‑miR-21 (**A**). Proliferation was measured by MTT assay 72 hours following the plating of transfected cells described above (n = 3) (**B**). Error bars represent standard error. * p < 0.001.

The observation that miR-21 expression is increased in primary malignant melanoma tumors led us to explore the function of miR-21 in human melanoma cell lines (WM1552c, WM793b, A375, and MEL 39). In order to examine the contribution of increased miR-21 expression to an aggressive melanoma phenotype, four melanoma cell lines were transfected with a non-specific control pre-miR or pre-miR-21. Mature miR-21 expression was significantly and consistently over-expressed in each cell line transfected with pre-miR-21 as compared to control pre-miR-transfected cell lines (WM1552c: 60.5 ± 32.6 fold increase; WM793b: 12.8 ± 4.5 fold increase; A375: 40.0 ± 13.2 fold increase; MEL 39: 100.2 ± 46.2 fold increase; all p < 0.001) (Fig. 1A).

Potential changes in the proliferative capacity of cells expressing elevated amounts of miR-21 were evaluated through mitochondrial reduction of yellow MTT to purple formazan forty-eight hours following the plating of transfected cells. Proliferation of pre-miR-21-transfected as compared to control pre-miR-transfected cells failed to produce an increase or decrease of greater than 10.8% in each of the four melanoma cell lines (Fig. 1B).

### Increased miR-21 activity leads to increased invasion but not migration *in vitro*

**Figure 2:**
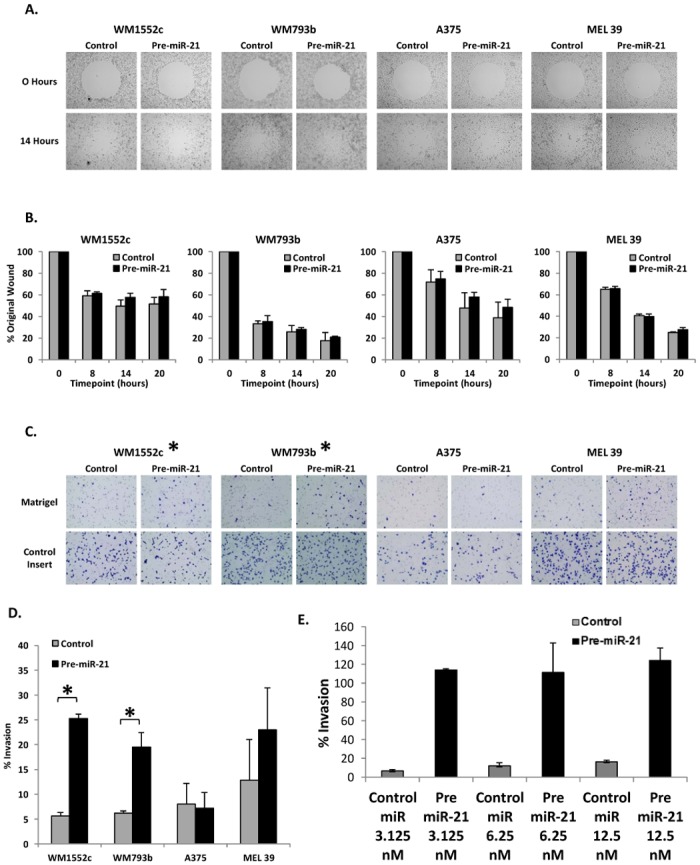
Migration assay wound closure following transfection with pre-miR-21 is consistent with control results. Following transfection with control pre-miR or pre-miR-21, cells were plated on Radius Migration Assay ECM-coated plates that have uniform wounds. Photographs were taken of the wound immediately following migration initiation and 14 hours later. Photographs of representative experiments for each cell line on the collagen-coated wells are shown (**A**). Migration was measured as the percent of the original wound area on the collagen-coated wells (n = 4) (**B**). Boyden chamber assays were used to evaluate invasive activity of melanoma cells transfected with control pre‑miR or pre-miR-21. Photographs of representative experiments for each cell line are shown (**C**). Data are represented as the ratio of the average number of cells migrating through control inserts to the average number of cells invading through inserts coated with matrigel (n = 4) (**D**). Following transfection with control pre-miR or pre-miR-21 at 3.125, 6.25, or 12.5 nM, cells were plated on Boyden chamber assays to evaluate invasive activity of A375 melanoma cells at lower doseage of oligonucleotide. Data are represented as the ratio of the average number of cells migrating through control inserts to the average number of cells invading through inserts coated with matrigel (**E**). Error bars represent standard error. * p < 0.05.

For metastasis to occur, primary melanoma cells must degrade the basement membrane and extracellular matrix and migrate through the stroma. In order to assess the effects of elevated miR-21 expression on melanoma cell movement, each of the four cell lines was transfected with pre-miR-21 or the control and plated onto Radius Migration Assay ECM-coated plates that have uniform wounds in the cell monolayers. Changes in lesion area were monitored over 0 to 20 hours (Fig. 2A, S2 Fig.). The difference in wound closure between control pre-miR-transfected and pre‑miR‑21‑transfected monolayers was unremarkable in each of the collagen‑coated wells (Fig. 2B), fibronectin-coated wells (S2 Fig.), and on the uncoated wells (S2 Fig.).

Invasive potential was examined using the Boyden chamber assay (Fig. 2C). Two of the four melanoma cell lines tested exhibited increased invasion upon transfection with pre‑miR‑21 as compared to control pre-miR-transfected cells (WM1552c: 25.3% ± 0.9 v. 5.6% ± 0.7; WM793b: 19.5% ± 2.9 v. 6.2% ± 0.5; A375: 7.1% ± 3.2 v. 8.0% ± 4.2; MEL 39: 23.0% ± 8.4 v. 12.8% ± 8.2) (Fig. 2D). This increase was significant in WM1552c and WM793b (p < 0.05) and approached significance in MEL 39. Notably, there appeared to be an effect of miR-21 at lower doses of oligonucleotide in A375 cells (Fig. 2E).

### Changes in TIMP3 protein expression are associated with miR-21 expression

**Figure 3:**
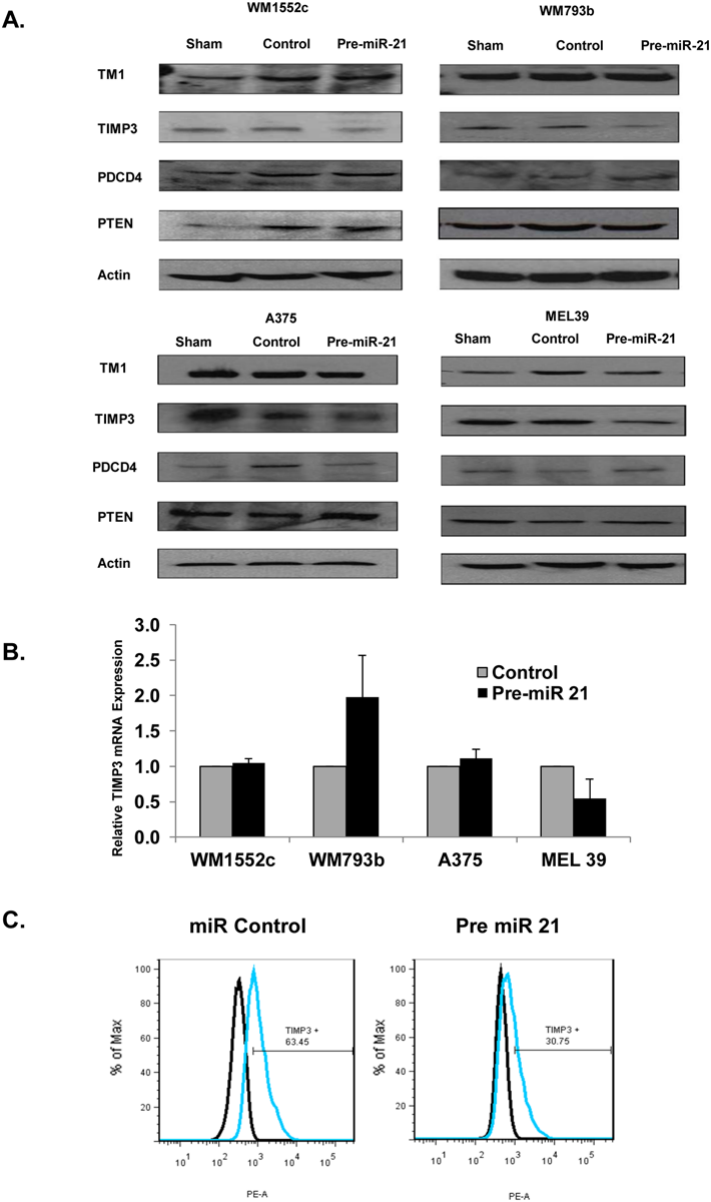
Changes in TIMP3 protein expression relate to miR-21 expression. Cells were collected 24 hours post-transfection for lysis, total RNA isolation, and flow cytometry. Twenty micrograms of protein were loaded and subjected to immunoblotting for the miR-21 putative target proteins TM1, TIMP3, PDCD4, and PTEN (**A**). Immunobloting for β-actin was used as the loading control. The RNA was converted to cDNA and Real‑Time PCR for TIMP3 was performed (**B**). Induction of expression was calculated relative to β-actin and compared to control-transfected cells. Cells were incubated with PE-anti-TIMP3 or isotype control Ab, washed and fixed in 1% formalin. A total of 10,000 cells were analyzed on a LSRII flow cytometer **(C)**. Error bars represent standard error.

Several mRNA targets for miR-21 have been recently verified, including phosphatase and tensin homolog (PTEN), tropomyosin-1 (TM1), and programmed cell death protein 4 (PCDC4) [20, 26–29]. Additionally, tissue inhibitor of metalloproteinases-3 (TIMP3) has been identified as a putative target and has been shown to be decreased in response to miR-21 [19, 20]. Immunoblots of cell lysates obtained 24 hours post‑transfection revealed a decrease in the TIMP3 protein in all four melanoma cell lines transfected with pre-miR-21 (Fig. 3A and S3 Fig.). There were no reproducible trends in the expression of TM1 and PCDC4. Interestingly, an increase in PTEN protein was observed in the WM1552c and A375 cell lines in response to pre-miR-21 transfection. Because miRs can achieve a decrease in protein expression by causing the degradation of target mRNAs and by inhibiting translation of mRNA, Real-Time PCR was performed to evaluate changes in TIMP3 mRNA expression in response to increased miR-21. TIMP3 mRNA expression was variable between transfection experiments and could not be associated with the consistently observed decrease in TIMP3 protein (Fig. 3B). Additionally, flow cytometric analysis of TIMP3 protein expression in the Mel 39 cell line following transfection with a pre-miR-21 construct showed a decrease in TIMP3 expression from 63.45% to 30.75% (Fig. 3C).

### Decreased TIMP3 expression increases melanoma invasiveness

**Figure 4:**
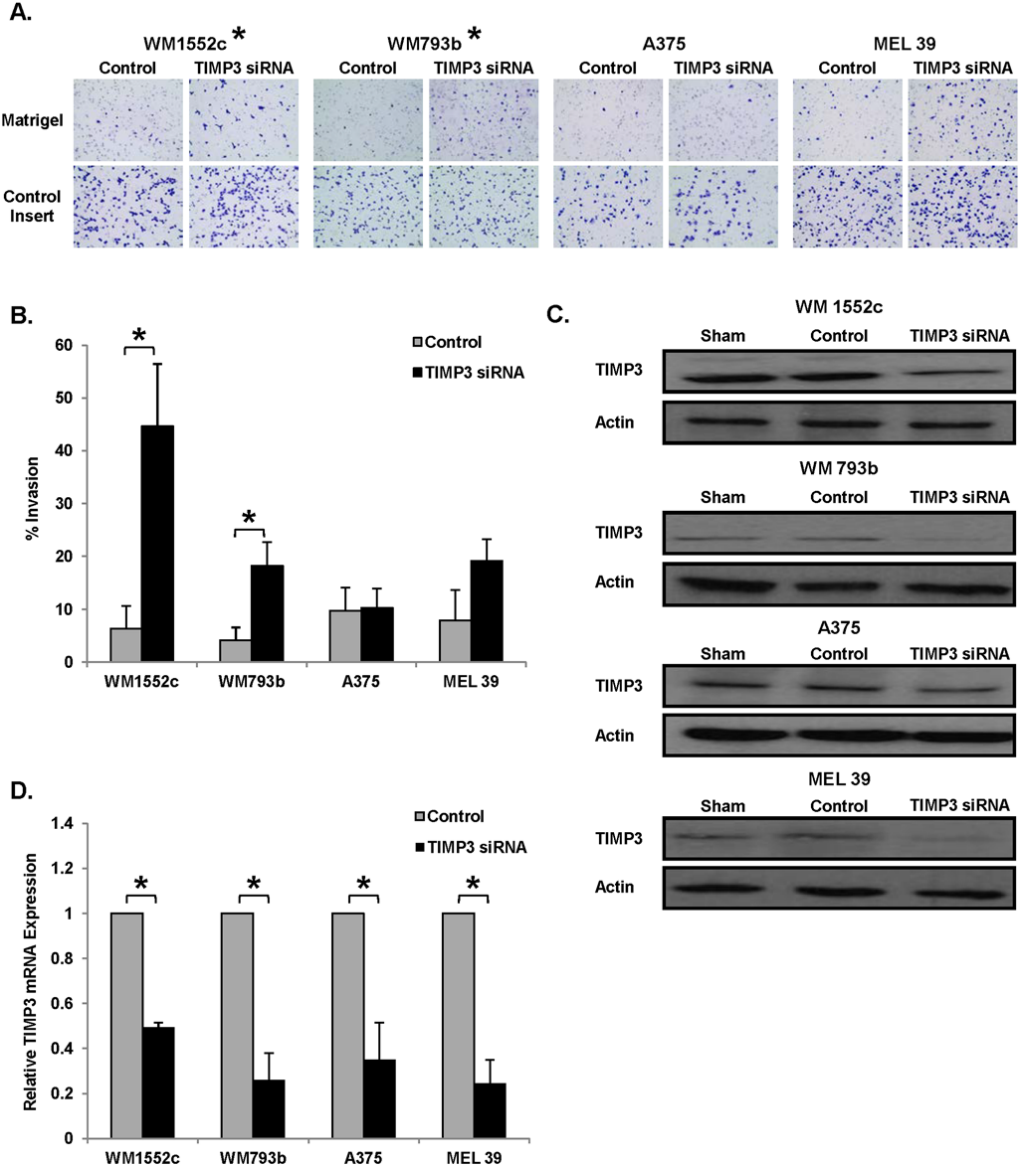
Invasion assays show that decreased TIMP3 expression increases melanoma invasiveness. Boyden chamber assays were used to evaluate invasive activity of melanoma cells transfected with negative control or TIMP3-specific siRNA. Photographs of representative experiments for each cell line are shown (**A**). Data are represented as the ratio of the average number of cells migrating through control inserts to the average number of cells invading through inserts coated with matrigel (n = 4) (**B**). Cells were also collected 24 hours post-transfection for lysis and total RNA isolation. Immunoblot performed on lysates confirmed down-regulation of TIMP3 expression (**C**). Immunoblotting for β-actin was used as the loading control. The RNA was converted to cDNA and Real-Time PCR for TIMP3 further confirmed down-regulation of TIMP3 expression (**D**). Error bars represent standard error. * p < 0.05.

The finding that TIMP3 expression was decreased in melanoma cells with elevated miR-21 and increased invasiveness prompted an exploration into the influence of TIMP3 on invasion. Invasive potential was examined using the Boyden chamber assay (Fig. 4A). Three of the four melanoma cell lines tested exhibited increased invasion when TIMP3 expression was down-regulated by siRNA as compared to the negative control‑transfected cells (WM1552c: 44.6% ± 11.8 v. 6.4% ± 4.3; WM793b: 18.2% ± 4.5 v. 4.2% ± 2.5; A375: 10.3% ± 3.5 v. 9.7% ± 4.3; MEL 39: 19.1% ± 4.1 v. 7.9% ± 5.7) (Fig. 4B). This increase was significant in WM1552c and WM793b (p < 0.05) and approached significance in MEL 39, mimicking the effects of miR-21 over-expression in cell lines. Reduction of TIMP3 transcript following siRNA transfection was confirmed by immunoblot (Fig. 4C) and Real-Time PCR (Fig. 4D).

### *In vitro* depletion of miR-21 in melanoma cells leads to decreased tumor size *in vivo*

**Figure 5:**
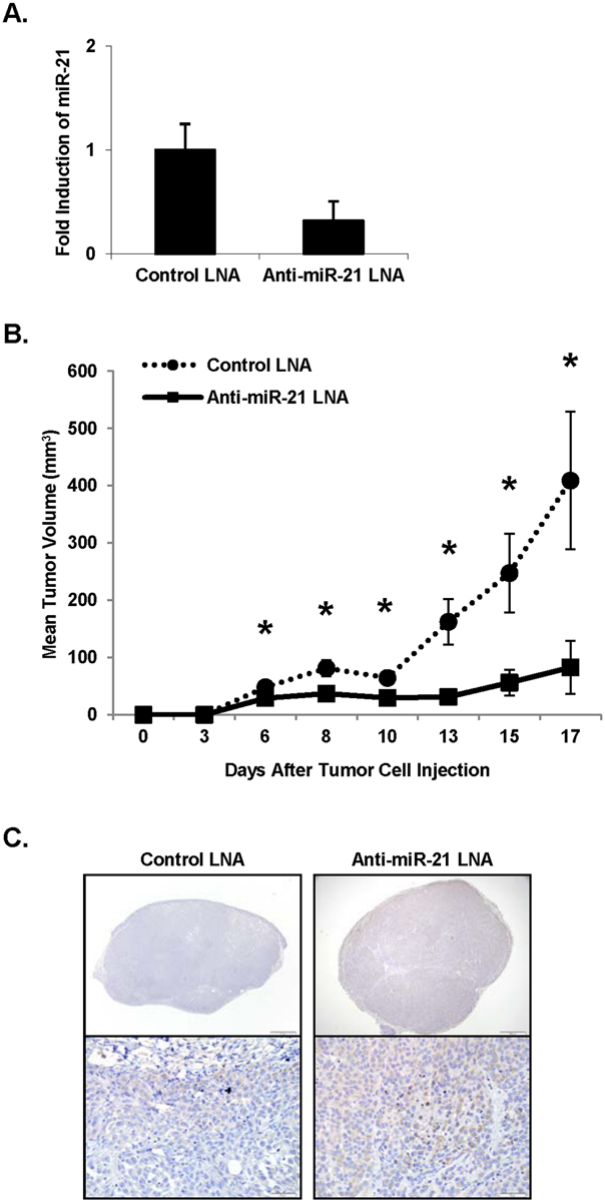
Decreased tumor size *in vivo* is achieved by depletion of miR-21 *in vitro*. A375 cells were transfected *in vitro* with an LNA oligonucleotide targeting miR-21 or a control LNA oligonucleotide. Cells were collected 24 hours post‑transfection for total RNA isolation. The RNA was converted to cDNA, and Real‑Time PCR for miR-21 confirmed reduced miR-21 expression (**A**). Transfected cells were then injected subcutaneously into athymic nude mice, and tumor growth was followed. Tumor volume was calculated as V = L × l^2^ × 0.5, where L and l represent the larger and the smaller tumor diameter, respectively. Mean tumor volumes are represented (**B**). At the completion of the study, tumors were harvested, fixed and embedded, and evaluated for TIMP3 protein by immunohistochemistry, where TIMP3 immunoreactivity is in brown (**C**). N = 4 for control LNA tumors and n = 6 for anti‑miR‑21 LNA tumors. Error bars represent standard error. * p < 0.05.

The data obtained demonstrating that miR-21 over-expression enhances invasion indicates that miR-21 may represent a treatment target. To test this hypothesis, A375 cells were transfected with an LNA oligonucleotide targeting miR-21 or a control LNA oligonucleotide. Real-Time PCR was performed on the cells 24 hours post-transfection and demonstrated reduced miR-21 expression in the cells transfected with anti-miR‑21 LNA (Fig. 5A). Notably, microRNA have been shown to resist degradation for up to three weeks through associations with target sequences, adenylation, and decreased uridylation [30]. Transfected cells were then injected subcutaneously into athymic nude mice, and tumor growth was followed. Mice injected with the anti-miR-21 LNA‑transfected cells developed significantly smaller tumors than those that received the control LNA-transfected cells. This difference became apparent beginning 6 days after tumor cell injection (p < 0.05) (Fig. 5B). At the completion of the study, tumors were harvested, fixed in formalin, embedded in paraffin, and evaluated for TIMP3 protein expression by immunohistochemistry. All control LNA-transfected tumors demonstrated 1+ staining for TIMP3, while the anti‑miR‑21 LNA-transfected tumors stained diffusely darker and exhibited 2+ staining on immunohistochemistry (Fig. 5C). Areas of confluent necrosis were negligible in both groups, and the mean mitotic indices were comparable in both groups (data not shown).

### The effects of i*n vivo* depletion of miR-21 on tumor size

**Figure 6:**
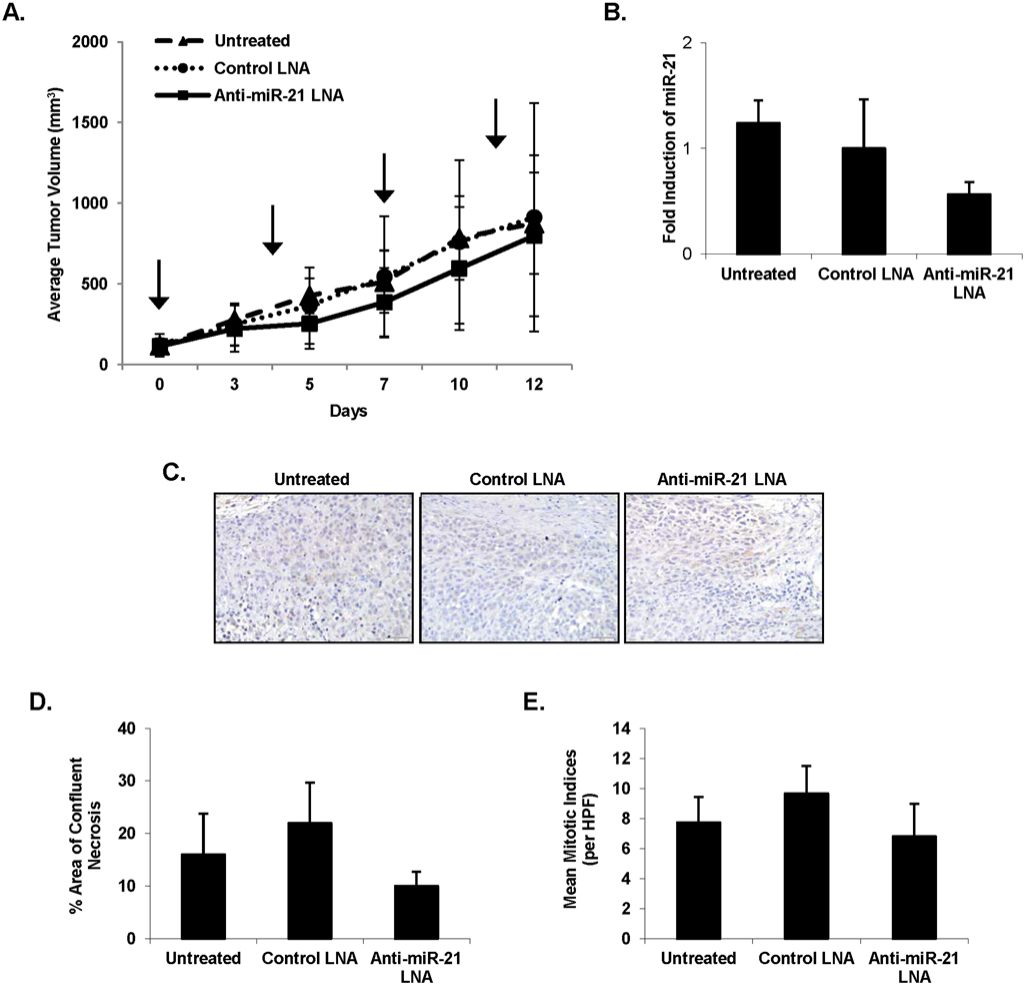
Evaluation of real time PCR and immunohistochemistry data from anti‑miR-21 LNA vs. control treated tumors. A375 cells were subcutaneously injected into the right flank of athymic nude mice and tumor development was measured. Each tumor was treated with four 50 µL injections of PBS, control LNA, or anti-miR-21 LNA at 500 nM at days 0, 4, 7, 11 (arrows). Day 0 is the day of the first treatment injection. Tumor volume was calculated as V = L × l^2^ × 0.5, where L and l represent the larger and the smaller tumor diameter, respectively. Mean tumor volumes are represented (**A**). Following the completion of the study, tumors were harvested. Total RNA was extracted from the tumors. The RNA was converted to cDNA, and Real-Time PCR for miR-21 confirmed reduced miR-21 expression (**B**). Tumors were also fixed, embedded, and evaluated for TIMP3 protein by immunohistochemistry, where TIMP3 immunoreactivity is in brown (**C**). Tumors were evaluated for mean areas of confluent necrosis (**D**) and mean mitotic indices (**E**). N = 5 for each group. Error bars represent standard error.

Encouraged by the previous results, the effect of direct *in vivo* anti-miR-21 LNA treatment on tumors was evaluated. Intra-tumoral delivery of an anti-miR was felt to be particularly relevant given the propensity of melanoma metastases to localize to skin and subcutaneous tissues. A375 cells were subcutaneously injected into the right flank of athymic nude mice and tumor development was followed. The tumors were then injected with PBS, the control LNA, or the anti-miR-21 LNA. There was a small decrease in tumor size for the group receiving anti-miR-21 treatments at days 5–10 but this difference was not appreciable by day 12 (Fig. 6A). Following the completion of the study, tumors were harvested. Total RNA was extracted from the tumors, and Real-Time PCR was performed in order ascertain whether the LNA injections had the predicted effect on the tumors. Real-Time PCR results demonstrated a reduction in miR-21 levels in the anti‑miR-21 LNA treated tumors as compared to the control LNA treated tumors (Fig. 6B). A portion of the tumors were fixed in formalin, embedded in paraffin, and evaluated for TIMP3 protein by immunohistochemistry (Fig. 6C). The untreated tumors demonstrated 1+ TIMP3 immunoreactivity in 4/5 tumors, and 1 to 2+ staining in 1/5 tumors. The control LNA-treated tumors had varying immunoreactivity, with no staining in 1/5 tumors, 1+ staining in 3/5 tumors, and 1 to 2+ staining in 1/5 tumors. The anti‑miR-21 LNA-treated tumors appeared to have increased levels of TIMP3 with 1+ staining in 2/5 tumors and 1 to 2+ staining in 3/5 tumors. Control LNA-treated tumors had larger mean areas of confluent necrosis (22%) as compared to anti-miR-21 LNA‑treated tumors (10%), although this did not reach statistical significance (p = 0.17) (Fig. 6D). The mean mitotic indices were decreased in the anti-miR-21 LNA-treated tumors (6.80) as compared to the control LNA-treated tumors (9.67), although this only approached significance (p = 0.09) (Fig. 6E).

## Discussion

These results demonstrate that increased miR-21 expression may enhance invasion and tumor growth, but not proliferation or migration, in multiple melanoma cell lines. Over-expression of miR‑21 resulted in a decrease in TIMP3 protein expression but did not alter that of several other recognized targets of miR-21. Importantly, miR-21 transfection of A375 melanoma cells did enhance invasion at lower doses of oligonucleotide thereby, supporting the use of A375 cells within our in vivo mouse studies. siRNA silencing of TIMP3 expression recapitulated the results obtained with miR-21 over-expression. Murine studies demonstrated that pre-treatment of melanoma cells with an anti-miR-21 reagent led to decreased tumor growth *in vivo*, but direct injection of tumors *in vivo* with an anti-miR-21 LNA only modestly inhibited tumor growth. Notably, the murine experiments confirmed that inhibition of miR-21 expression led to increased TIMP3 protein expression. The effects of miR-21 over-expression on melanoma invasion therefore appear to be mediated by down-regulation of TIMP3.

TIMP3 is a member of the tissue inhibitor of metalloproteinases family that binds metalloproteinases and other proteolytic enzymes to reduce their activity. Unlike other TIMPs, TIMP3 has decreased solubility and is found in association with the extracellular matrix and basement membrane and not in cell culture supernatants [7, 31, 32]. Cruz‑Munoz *et al.* demonstrated enhanced metastatic dissemination of melanoma cells to the lungs and bone in TIMP3^-/-^ mice [33]. While over-expression of TIMP3 in cell lines derived from cutaneous melanoma metastases has been shown to decrease invasion *in vitro* [34], this study represents the first time a miR-induced decrease in TIMP3 has been shown to confer an invasive phenotype on a cell line originating from a primary radial growth melanoma WM1552c, a form of melanoma that normally lacks the capacity to invade and metastasize.

During the initial radial growth phase, dysplastic melanocytes typically spread laterally within the epidermis. As the cells gain metastatic potential, they transition to the vertical growth phase, and acquire the ability to penetrate the basement membrane and increase vascularization [4]. In doing so, degradation of the ECM is necessary, and MMPs are often implicated in that process [5]. TIMP3 acts to inhibit MMPs, but its expression can be reduced in certain malignancies [7, 35]. This implicates a decrease in TIMP3 expression as a potential mechanism by which melanomas exhibiting only the radial growth phase may transition to the more aggressive vertical growth phase.

Little is known about how TIMP3 expression is regulated in melanoma tumorigenesis. Van der Velden *et al.* demonstrated that TIMP3 expression was regulated by methylation in cell lines and primary uveal melanomas [36]. However, promoter methylation in primary cutaneous melanomas and metastatic lesions was shown to be a low frequency event [37], suggesting that the decreased expression of TIMP3 in melanoma must be accomplished through another mechanism of regulation. Interestingly, a study of uveal melanomas observed high levels of TIMP3 mRNA transcripts in association with little to no expression of TIMP3 protein [38]. This supports the hypothesis that post-transcriptional regulation of TIMP3 is critical in melanoma. The findings that miR‑21 expression is elevated in primary melanoma tumors [18] and that miR-21 over-expression down-regulates TIMP3 protein expression implicates a role for miR-21 silencing of TIMP3 in the progression of melanoma.

While miR-21 has been identified as a microRNA commonly over-expressed in several solid tumors [39], the current study represents the first time a function has been ascribed to miR‑21 in the context of melanoma. miR-21 expression has been previously associated with an invasive phenotype in several malignancies due to its ability to target multiple pathways affecting this process. Meng *et al.* found miR-21-mediated increases in hepatocellular carcinoma invasion were due to the direct targeting of PTEN mRNA [26]. The resulting decrease in PTEN protein was associated with enhanced expression of MMP-2 and MMP-9, two matrix proteases that are also implicated in melanoma pathogenesis. A role for miR-21-associated down-regulation of PDCD4 in colon cancer and TM1 and PDCD4 in breast cancer has also been confirmed [27, 28]. In the current study, however, increased miR-21 expression was not found to decrease PTEN, TM1, or PDCD4 protein 24 hours post-transfection, suggesting that these pathways may not play a major role in miR-21-initiated invasion of melanoma. Other newly identified targets of miR‑21, including Maspin, MARCKS, and RECK, have been shown to participate in decreasing cancer cell invasion through matrigel and therefore warrant exploration into their ability to do so in melanoma [19, 27, 40, 41].

The observation of a greater invasive potential without a concomitant increase in migration in miR-21-over-expressing cells suggests that miR-21 may be acting on one or more factors affecting the cell’s ability to degrade the extracellular matrix after they have already acquired increased mobility. This migration assay examined the cells’ ability to migrate through individual ECM proteins (e.g. collagen and fibronectin), but it may be that additional factors within the native ECM are required for miR-21 over‑expressing melanoma cells to exhibit increased migration.

LNAs were used to achieve reduced expression of miR-21 within tumors *in vivo*. Tumors of cells transfected pre-implantation with the anti-miR-21 LNA had significantly decreased tumor growth and increased TIMP3 protein expression. However, intra‑tumoral injections of anti-miR-21 into established tumors only modestly inhibited tumor growth, but did appear to have some effect on tumor necrosis. Likewise, the change in TIMP3 protein expression was not as definitive when miR-21 expression was modulated via intra-tumoral injections of LNAs. These results indicate that miR-21 may have a role in melanoma tumor formation; however, the down-stream effects of miR-21 over-expression may not be easily reversible via intra-tumoral injections of currently available LNA constructs.

These results suggest that regulators of TIMP3 can lead to altered tumor growth *in vivo* independent of cellular proliferation. These findings differ from previous findings which suggest an involvement of miR-21 in mitotic rate [18]. Interestingly, reduced tumor volume may be attributed to modified cellular density within the tissue as suggested by Storz *et al.* who showed that the FOXO3a gene was responsible for increased invasion of breast carcinoma cells and that inhibition of this gene in vitro with a shRNA led to reduced invasiveness and a less dense tumor volume [42]. Notably, *in vivo* studies of FOXO3a inhibition in a breast tumor model revealed reduced tumor size as compared to controls, thus providing support for our contention that inhibition of genes involved in cellular invasion may also result in reduced tumor volume [42]. Similar results have been obtained for melanoma tumors as well where TIMP3 down-regulation by shRNA promoted angiogenesis and increased tumor size [43].

Decreased expression of TIMP3 in response to increased miR-21 was first described in cholangiocarcinoma and glioma [19, 20]. Gabriely *et al.* demonstrated that glioma cells transfected with an anti‑miR‑21 construct exhibited a consistent increase in TIMP3 mRNA and protein expression. While they carefully examined the effects of miR-21 antagonism on MMP activity and cellular invasion, they did not explore the effect of TIMP3 siRNA on invasion, as was done in this study [19]. Selaru *et al.* elegantly demonstrated that miR-21 is elevated in cholangiocarcinoma tissue samples and miR-21 inhibition increases TIMP3 protein expression in cholangiocarcinoma cell lines, but they did not explore the effects of miR‑21 over-expression on cell line behavior, and results were not confirmed in a murine model [20]. Wang *et al.* showed that miR-21 alters cellular invasion in a TIMP3 dependent manner in the setting of esophageal carcinoma while Zhang *et al.* showed a similar effect in renal cell carcinoma [44, 45]. However, this effect has not been previously described in the context of melanoma. In the current study, the effects of miR-21 over-expression were documented in multiple cell lines. The levels of miR-21 and TIMP3 were evaluated by Real-Time PCR, and TIMP3 protein levels were examined by immunoblot as well. Importantly, the increased invasion of miR-21 over-expressing cells was reproduced in TIMP3 siRNA‑transfected cells. Finally, this study evaluated the ability of miR-21 inhibition to exert anti-melanoma effects *in vivo*.

There are pitfalls of this study that deserve attention. Notably, one must consider the potential off-target effects of miR-21 when evaluating the role of TIMP3 reductions on the invasion capacity of melanoma cells. There is always the possibility that other genes regulated by miR-21 could mediate an effect on invasion. miR-21 has numerous targets involved in a myriad of cellular processes and while our efforts focused on commonly regulated genes, it is plausible that unstudied genes may have also contributed to the differences in cellular invasion. Furthermore, miR control constructs contain random, non-specific sequences, and it is possible that these constructs may inadvertently harbor biologic activity which could undermine the findings herein. Therefore, interpretation of the effects of miR-21 on TIMP3 protein levels must be tempered by the realization that the miR control constructs could exert some effect on TIMP3 levels.

This study is the first to identify TIMP3 as a potential target of miR-21 in the context of melanoma and demonstrates that down-regulation of TIMP3 may lead to increased melanoma invasion. Furthermore, miR-21 might be a regulator of tumor growth and this effect may be dependent on TIMP3.

## Supporting Information

S1 Fig Transfection Efficiency of Melanoma Cells. A375 cells were transfected with a FAM-conjugated control miR (green) construct at a concentration of 25 nM. Cells were harvested after incubation overnight and counterstained with DAPI (blue) before visualization via fluorescent microscopy. Transfection efficiency was calculated as the ratio of FAM-positive cells to DAPI-positive cells. This analysis was repeated using the WM 793, WM 1552c, and Mel 39 cell lines and similar results were obtained.
(TIF)

S2 Fig Wound closure in migration assays at T = 0 and T = 14 hours. Following transfection with control pre-miR or pre-miR-21, cells were plated on Radius Migration Assay ECM-coated plates that have uniform wounds. Photographs were taken of the wound immediately following migration initiation and 14 hours later. Photographs of representative experiments for each cell line are shown. Migration was measured as the percent of the original wound area on the fibronectin-coated (n = 4) and uncoated wells (n = 4) for WM1552c (**A**), WM793b (**B**), A375 (**D**), and Mel39 (**D**) melanoma cells. Error bars represent standard error.
(TIF)

S3 Fig Western blot analysis of TIMP3 expression following transfection with a pre-miR-21 construct. WM793, Mel 39, A375, and WM 1552 melanoma cells were transfected with pre-miR-21 or a control miR (25 nM) and incubated overnight prior to harvesting. Immunobloting for TIMP3 was performed with anti-TIMP3 or anti-β-Actin antibody. Quantification was performed using image J software. Expression was normalized to β-Actin and expressed relative to untransfected cells which was designated as 1.00.
(TIF)

## References

[1] Siegel R, Naishadham D, Jemal A (2013) Cancer statistics, CA Cancer J Clin. 2013; 63:11–30. 10.3322/caac.21166 23335087

[2] Horner M, Ries L, Krapcho M, Neyman N, Aminou R, et al (2009) Surveillance Epidemiology and End Result (SEER) Cancer Statistics Review, 1975–2006. Available from: http://seer.cancer.gov/csr/1975_2006/ - contents. Accessed 10 December 2014.

[3] Coit DG, Andtbacka R, Anker CJ, Bichakjian CK, Carson WE, et al (2012) Melanoma. J Natl Compr Canc Netw. 10:366–400. 22393197 10.6004/jnccn.2012.0036

[4] Gaggioli C, Sahai E (2007) Melanoma invasion - current knowledge and future directions. Pigment Cell Res. 20:161–72. 10.1111/j.1600-0749.2007.00378.x 17516924

[5] Ahonen M, Poukkula M, Baker AH, Kashiwagi M, Nagase H, et al (2003) Tissue inhibitor of metalloproteinases-3 induces apoptosis in melanoma cells by stabilization of death receptors. Oncogene. 22:2121–34. 10.1038/sj.onc.1206292 12687014

[6] Hofmann UB, Houben R, Bröcker EB, Becker JC (2005) Role of matrix metalloproteinases in melanoma cell invasion. Biochimie. 87:307–14. 10.1016/j.biochi.2005.01.013 15781317

[7] Zeng Z, Sun Y, Shu W, Guillem JG (2001) Tissue inhibitor of metalloproteinase-3 is a basement membrane-associated protein that is significantly decreased in human colorectal cancer. Dis Colon Rectum. 44:1290–6. 10.1007/BF02234786 11584202

[8] Ahonen M, Poukkula M, Baker AH, Kashiwagi M, Nagase H, et al (2003) Tissue inhibitor of metalloproteinases-3 induces apoptosis in melanoma cells by stabilization of death receptors. Oncogene. 22:2121–34. 10.1038/sj.onc.1206292 12687014

[9] Hu S, Liu D, Tufano RP, Carson KA, Rosenbaum E, et al (2006) Association of aberrant methylation of tumor suppressor genes with tumor aggressiveness and BRAF mutation in papillary thyroid cancer. Int J Cancer. 119:2322–9. 10.1002/ijc.22110 16858683

[10] Jiang X, Huang X, Li J, Shi Y, Zhou L (2000) Relationship between tissue inhibitors of metalloproteinase and metastasis and prognosis in breast cancer. Zhonghua Wai Ke Za Zhi. 38:291–3, 19 12828172

[11] Karan D, Lin FC, Bryan M, Ringel J, Moniaux N, et al (2003) Expression of ADAMs (a disintegrin and metalloproteases) and TIMP-3 (tissue inhibitor of metalloproteinase-3) in human prostatic adenocarcinomas. Int J Oncol. 23:1365–71. 14532978

[12] Kettunen E, Anttila S, Seppänen JK, Karjalainen A, Edgren H, et al (2004) Differentially expressed genes in nonsmall cell lung cancer: expression profiling of cancer-related genes in squamous cell lung cancer. Cancer Genet Cytogenet. 149:98–106. 10.1016/S0165-4608(03)00300-5 15036884

[13] Feng H, Cheung AN, Xue WC, Wang Y, Wang X, et al (2004) Down-regulation and promoter methylation of tissue inhibitor of metalloproteinase 3 in choriocarcinoma. Gynecol Oncol. 94:375–82. 10.1016/j.ygyno.2004.04.019 15297175

[14] Bartel DP (2004) MicroRNAs: genomics, biogenesis, mechanism, and function. Cell. 116:281–97. 10.1016/S0092-8674(04)00045-5 14744438

[15] He L, Hannon GJ (2004) MicroRNAs: small RNAs with a big role in gene regulation. Nat Rev Genet. 5:522–31. 10.1038/nrg1379 15211354

[16] Dalmay T, Edwards DR (2006) MicroRNAs and the hallmarks of cancer. Oncogene. 25:6170–5. 10.1038/sj.onc.1209911 17028596

[17] Lu J, Getz G, Miska EA, Alvarez-Saavedra E, Lamb J, et al (2005) MicroRNA expression profiles classify human cancers. Nature. 435:834–8. 10.1038/nature03702 15944708

[18] Grignol V, Fairchild ET, Zimmerer JM, Lesinski GB, Walker MJ, et al (2011) miR-21 and miR-155 are associated with mitotic activity and lesion depth of borderline melanocytic lesions. Br J Cancer. 105:1023–9. 10.1038/bjc.2011.288 21863027 PMC3185929

[19] Gabriely G, Wurdinger T, Kesari S, Esau CC, Burchard J, et al (2008) MicroRNA 21 promotes glioma invasion by targeting matrix metalloproteinase regulators. Mol Cell Biol. 28:5369–80. 10.1128/MCB.00479-08 18591254 PMC2519720

[20] Selaru FM, Olaru AV, Kan T, David S, Cheng Y, et al (2009) MicroRNA-21 is overexpressed in human cholangiocarcinoma and regulates programmed cell death 4 and tissue inhibitor of metalloproteinase 3. Hepatology. 49:1595–601. 10.1002/hep.22838 19296468 PMC3124086

[21] Song B, Wang C, Liu J, Wang X, Lv L, et al (2010) MicroRNA-21 regulates breast cancer invasion partly by targeting tissue inhibitor of metalloproteinase 3 expression. J Exp Clin Cancer Res. 29:29 10.1186/1756-9966-29-29 20346171 PMC2853500

[22] Satyamoorthy K, DeJesus E, Linnenbach AJ, Kraj B, Kornreich DL, et al (1997) Melanoma cell lines from different stages of progression and their biological and molecular analyses. Melanoma Res. 7 Suppl 2:S35–42. 9578415

[23] Lesinski GB, Trefry J, Brasdovich M, Kondadasula SV, Sackey K, et al (2007) Melanoma cells exhibit variable signal transducer and activator of transcription 1 phosphorylation and a reduced response to IFN-alpha compared with immune effector cells. Clin Cancer Res. 13:5010–9. 10.1158/1078-0432.CCR-06-3092 17785551

[24] Schmittgen TD, Livak KJ (2008) Analyzing real-time PCR data by the comparative C(T) method. Nat Protoc. 3:1101–8. 10.1038/nprot.2008.73 18546601

[25] Srinivasan R, Zabuawala T, Huang H, Zhang J, Gulati P, et al (2009) Erk1 and Erk2 regulate endothelial cell proliferation and migration during mouse embryonic angiogenesis. PLoS One. 4:e8283 10.1371/journal.pone.0008283 20011539 PMC2789384

[26] Meng F, Henson R, Wehbe-Janek H, Ghoshal K, Jacob ST, et al (2007) MicroRNA-21 regulates expression of the PTEN tumor suppressor gene in human hepatocellular cancer. Gastroenterology. 133:647–58. 10.1053/j.gastro.2007.05.022 17681183 PMC4285346

[27] Zhu S, Wu H, Wu F, Nie D, Sheng S, et al (2008) MicroRNA-21 targets tumor suppressor genes in invasion and metastasis. Cell Res. 18:350–9. 10.1038/cr.2008.24 18270520

[28] Asangani IA, Rasheed SA, Nikolova DA, Leupold JH, Colburn NH, et al (2008) MicroRNA-21 (miR-21) post-transcriptionally downregulates tumor suppressor Pdcd4 and stimulates invasion, intravasation and metastasis in colorectal cancer. Oncogene. 27:2128–36. 10.1038/sj.onc.1210856 17968323

[29] Frankel LB, Christoffersen NR, Jacobsen A, Lindow M, Krogh A, et al (2008) Programmed cell death 4 (PDCD4) is an important functional target of the microRNA miR-21 in breast cancer cells. J Biol Chem. 283:1026–33. 10.1074/jbc.M707224200 17991735

[30] Zhang Z, Qin Y, Brewer G, Jing Q (2012) MicroRNA degradation and turnover: Regulating the Regulators. WIREs RNA 3:593–600. 10.1002/wrna.1114 22461385 PMC3635675

[31] Leco KJ, Khokha R, Pavloff N, Hawkes SP, Edwards DR (1994) Tissue inhibitor of metalloproteinases-3 (TIMP-3) is an extracellular matrix-associated protein with a distinctive pattern of expression in mouse cells and tissues. J Biol Chem. 269:9352–60. 8132674

[32] Yu WH, Yu S, Meng Q, Brew K, Woessner JF (2000)TIMP-3 binds to sulfated glycosaminoglycans of the extracellular matrix. J Biol Chem. 275:31226–32. 10.1074/jbc.M000907200 10900194

[33] Cruz-Munoz W, Sanchez OH, Di Grappa M, English JL, Hill RP, et al (2006) Enhanced metastatic dissemination to multiple organs by melanoma and lymphoma cells in timp-3-/- mice. Oncogene. 25:6489–96. 10.1038/sj.onc.1209663 16702949

[34] Ahonen M, Baker AH, Kahari VM (1998) Adenovirus-mediated gene delivery of tissue inhibitor of metalloproteinases-3 inhibits invasion and induces apoptosis in melanoma cells. Cancer Res. 58:2310–5. 9622064

[35] Jiang X, Huang X, Li J, Shi Y, Zhou L (2000) Relationship between tissue inhibitors of metalloproteinase and metastasis and prognosis in breast cancer. Zhonghua Wai Ke Za Zhi. 38:291–3, 19 12828172

[36] van der Velden PA, Zuidervaart W, Hurks MH, Pavey S, Ksander BR, et al (2003) Expression profiling reveals that methylation of TIMP3 is involved in uveal melanoma development. Int J Cancer. 106:472–9. 10.1002/ijc.11262 12845640

[37] Liu S, Ren S, Howell P, Fodstad O, Riker AI (2008) Identification of novel epigenetically modified genes in human melanoma via promoter methylation gene profiling. Pigment Cell Melanoma Res. 21:545–58. 10.1111/j.1755-148X.2008.00484.x 18627528

[38] Nareyeck G, Zeschnigk M, von der Haar D, Schilling H, Bornfeld N, et al (2005) Differential expression of tissue inhibitor of matrix metalloproteinases 3 in uveal melanoma. Ophthalmic Res. 37:23–8. 10.1159/000082940 15637418

[39] Volinia S, Calin GA, Liu CG, Ambs S, Cimmino A, et al (2006) A microRNA expression signature of human solid tumors defines cancer gene targets. Proc Natl Acad Sci U S A. 103:2257–61. 10.1073/pnas.0510565103 16461460 PMC1413718

[40] Zhang Z, Li Z, Gao C, Chen P, Chen J, et al (2008) miR-21 plays a pivotal role in gastric cancer pathogenesis and progression. Lab Invest. 88:1358–66. 10.1038/labinvest.2008.94 18794849

[41] Li T, Li D, Sha J, Sun P, Huang Y (2009) MicroRNA-21 directly targets MARCKS and promotes apoptosis resistance and invasion in prostate cancer cells. Biochem Biophys Res Commun. 383:280–5. 10.1016/j.bbrc.2009.03.077 19302977

[42] Storz P, Doppler H, Copland JA, Simpson KJ, Toker A (2009) FOXO3a Promotes Tumor Cell Invasion through the Induction of Matrix Metalloproteinases. Mol Cell Biol. 29: 4906–4917. 10.1128/MCB.00077-09 19564415 PMC2738298

[43] Das AM, Seynhaeve ALB, Rens JAP, Vermeulen CE, Koning GA, et al (2014) Differential TIMP3 expression affects tumor progression and angiogenesis in melanomas through regulation of directionally persistent endothelial cell migration. Angiogenesis 17:163–177. 10.1007/s10456-013-9385-2 24221409

[44] Wang N, Zhang C, He J, Duan X, Wang Y, et al (2013) miR-21 Down-Regulation Suppresses Cell Growth, Invasion and Induces Cell Apoptosis by Targeting FASL, TIMP3, and RECK Genes in Esophageal Carcinoma. Dig Dis Sci. 58:1863–1870. 10.1007/s10620-013-2612-2 23504349

[45] Zhang A, Liu Y, Shen Y, Xu Y, Li X (2011) miR-21 Modulates Cell Apoptosis by Targeting Multiple Genes in Renal Cell Carcinoma. Urology 78: 474.e13– 474.e19. 10.1016/j.urology.2011.03.030 21820586

