# Supplementary figures and images for: MiR-21 Enhances Melanoma Invasiveness via Inhibition of Tissue Inhibitor of Metalloproteinases 3 Expression: In Vivo Effects of MiR-21 Inhibitor
Source: PLoS One. 2015 Jan 14;10(1):e0115919. doi: 10.1371/journal.pone.0115919 (PMC4294659; doi:10.1371/journal.pone.0115919)

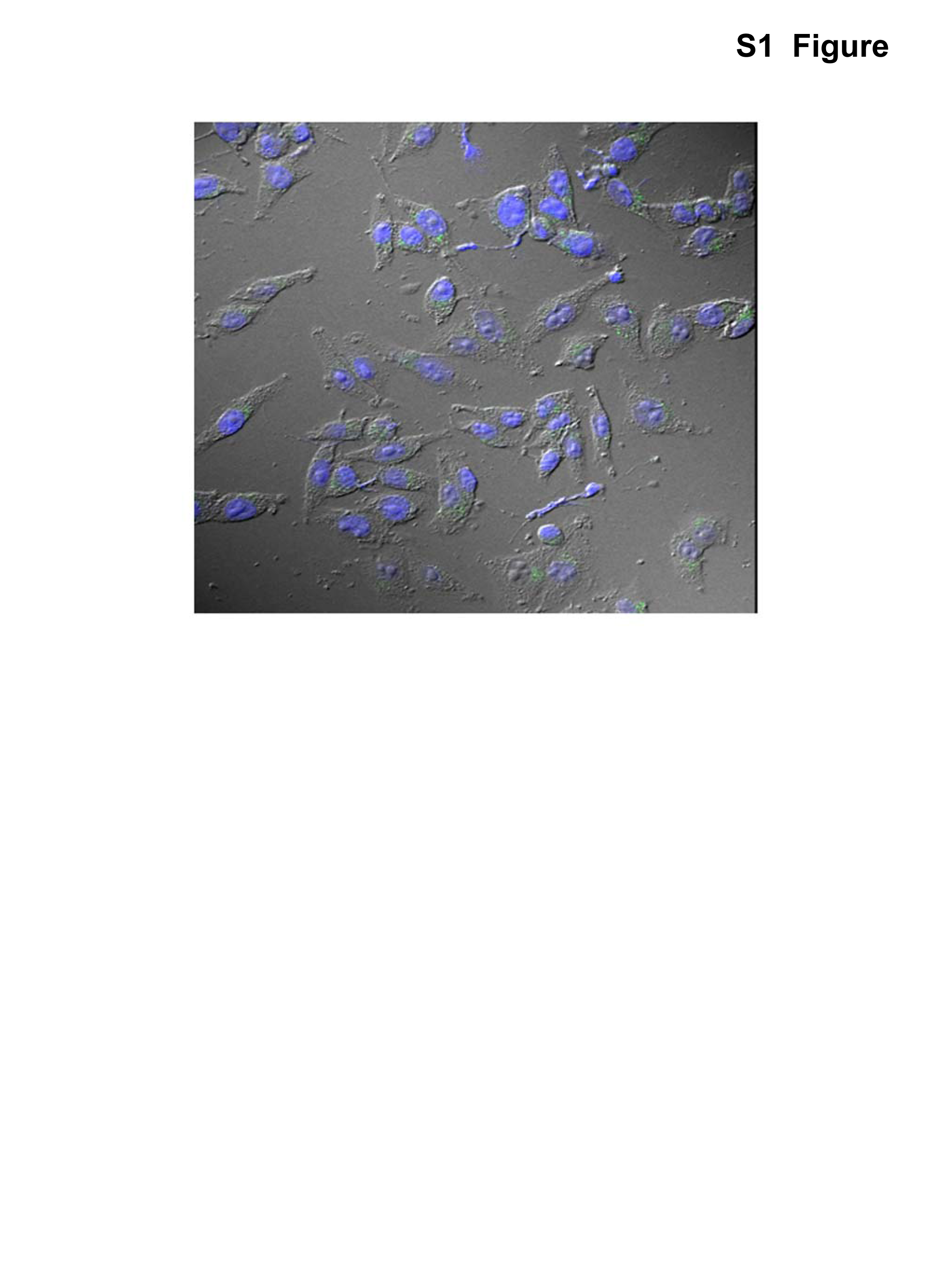

Supplement: S1 Fig — A375 cells were transfected with a FAM-conjugated control miR (green) construct at a concentration of 25 nM. Cells were harvested after incubation overnight and counterstained with DAPI (blue) before visualization via fluorescent microscopy. Transfection efficiency was calculated as the ratio of FAM-positive cells to DAPI-positive cells. This analysis was repeated using the WM 793, WM 1552c, and Mel 39 cell lines and similar results were obtained. (TIF) [file pone.0115919.s001.tif]

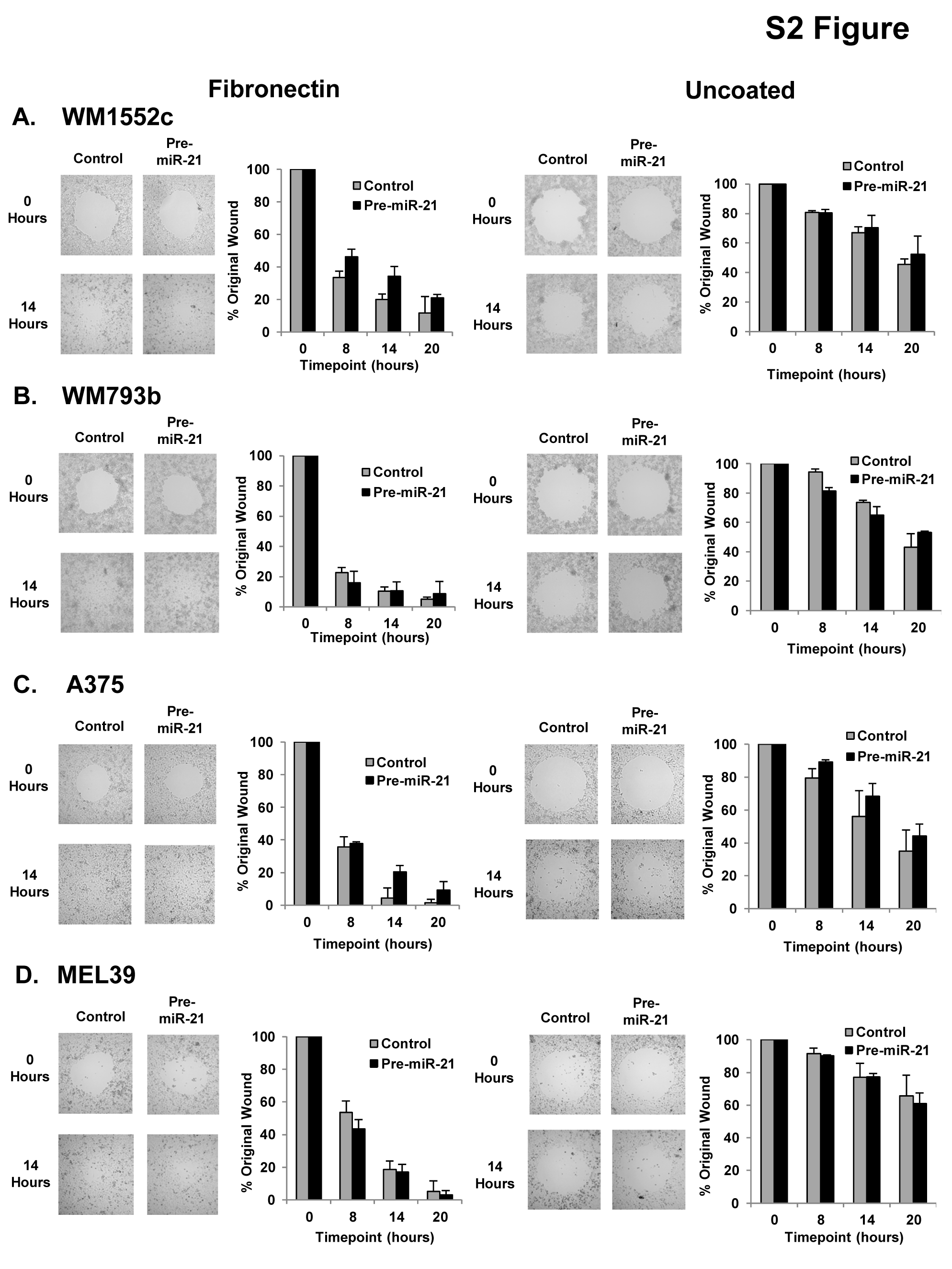

Supplement: S2 Fig — Following transfection with control pre-miR or pre-miR-21, cells were plated on Radius Migration Assay ECM-coated plates that have uniform wounds. Photographs were taken of the wound immediately following migration initiation and 14 hours later. Photographs of representative experiments for each cell line are shown. Migration was measured as the percent of the original wound area on the fibronectin-coated (n = 4) and uncoated wells (n = 4) for WM1552c (A), WM793b (B), A375 (D), and Mel39 (D) melanoma cells. Error bars represent standard error. (TIF) [file pone.0115919.s002.tif]

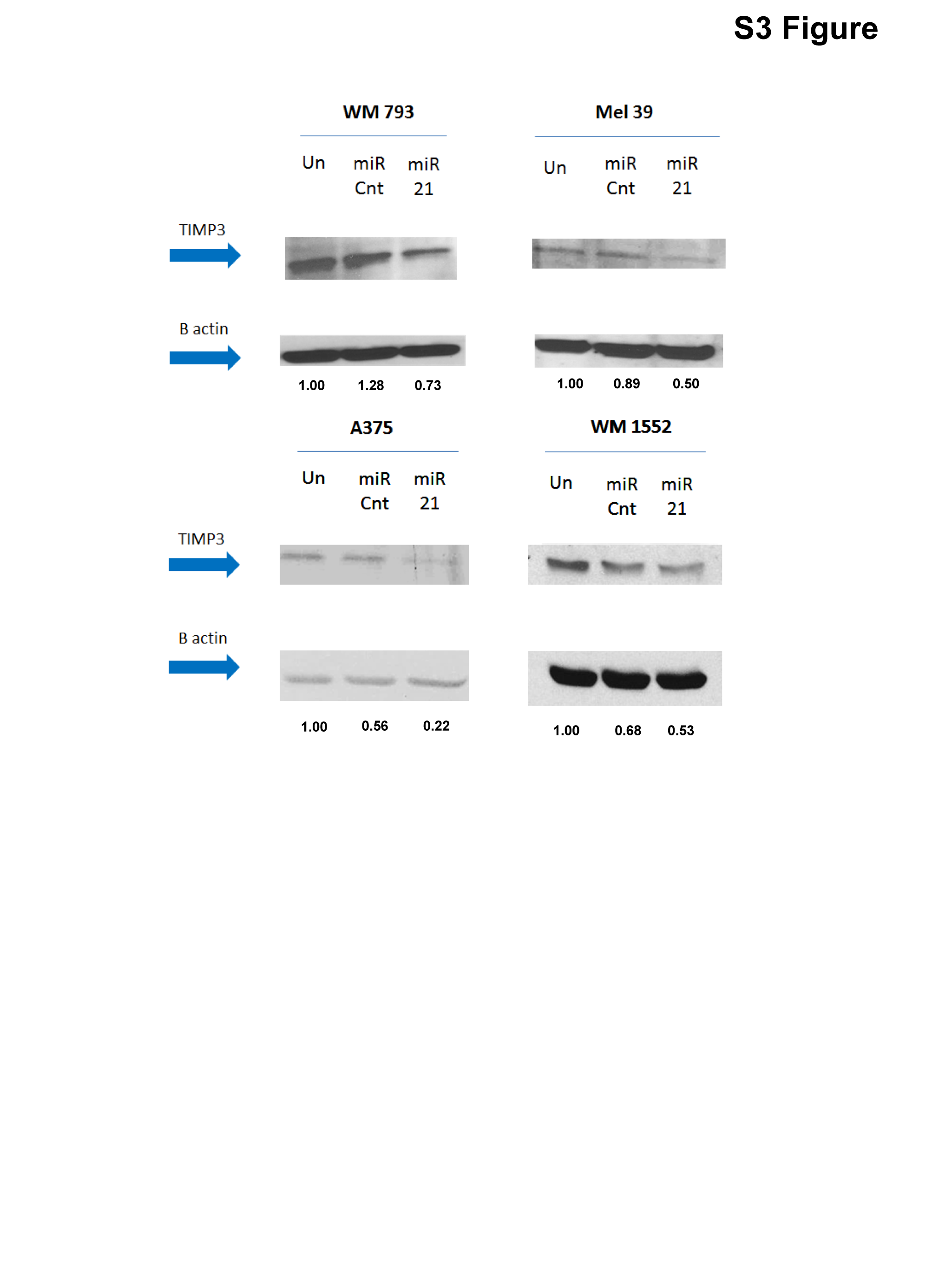

Supplement: S3 Fig — WM793, Mel 39, A375, and WM 1552 melanoma cells were transfected with pre-miR-21 or a control miR (25 nM) and incubated overnight prior to harvesting. Immunobloting for TIMP3 was performed with anti-TIMP3 or anti-β-Actin antibody. Quantification was performed using image J software. Expression was normalized to β-Actin and expressed relative to untransfected cells which was designated as 1.00. (TIF) [file pone.0115919.s003.tif]
